# Five-Year Follow-Up After Transcatheter Aortic Valve Implantation in Patients with Severe Aortic Stenosis and Concomitant Coronary Artery Disease: A Single-Center Experience

**DOI:** 10.21470/1678-9741-2022-0461

**Published:** 2023-10-23

**Authors:** Akram Abawi, Anders Magnuson, Ole Fröbert, Ninos Samano

**Affiliations:** 1 Department of Radiology, Örebro University Hospital, Örebro, Sweden; 2 Clinical Epidemiology and Biostatistics, School of Medical Sciences, Faculty of Medicine and Health, Örebro University, Örebro, Sweden; 3 Department of Cardiology, Faculty of Medicine and Health, Örebro University, Örebro, Sweden; 4 University Health Care Research Centre, Faculty of Medicine and Health, Örebro University, Örebro, Sweden

**Keywords:** Aortic Stenosis, Coronary Artery Bypass Grafting, Coronary Artery Disease, Percutaneous Coronary Intervention, Transcatheter Aortic Valve Implantation

## Abstract

**Introduction:**

There is no consensus on the impact of coronary artery disease in patients undergoing transcatheter aortic valve implantation. Therefore, the objective of this study was, in a single-center setting, to evaluate the five-year outcome of transcatheter aortic valve implantation patients with or without coronary artery disease.

**Methods:**

All transcatheter aortic valve implantation patients between 2009 and 2019 were included and grouped according to the presence or absence of coronary artery disease. The primary endpoint, five-year all-cause mortality, was evaluated using Cox regression adjusted for age, sex, procedure years, and comorbidities. Comorbidities interacting with coronary artery disease were evaluated with interaction tests. In-hospital complications was the secondary endpoint.

**Results:**

In total, 176 patients had aortic stenosis and concomitant coronary artery disease, while 170 patients had aortic stenosis only. Mean follow-up was 2.2±1.6 years. There was no difference in the adjusted five-year all-cause mortality between transcatheter aortic valve implantation patients with and without coronary artery disease (hazard ratio 1.00, 95% confidence interval 0.59-1.70, P=0.99). In coronary artery disease patients, impaired renal function, peripheral arterial disease, or ejection fraction < 50% showed a significant interaction effect with higher five-year all-cause mortality. No significant differences in complications between the groups were found.

**Conclusion:**

Five-year mortality did not differ between transcatheter aortic valve implantation patients with or without coronary artery disease. However, in patients with coronary artery disease and impaired renal function, peripheral arterial disease, or ejection fraction < 50%, we found significantly higher five-year all-cause mortality.

## INTRODUCTION

Severe aortic stenosis (AS) is a common condition among the elderly, with a prevalence of 3.4% in patients > 75 years old^[1]^. With medical treatment only, the condition carries a poor prognosis, with a reported three-year all-cause mortality reported at 57% by one study^[2]^. Surgical aortic valve replacement (AVR) was the only available treatment in the past and has been shown to be superior to medical therapy even in asymptomatic patients^[3]^. Since it was first performed by Alain Cribier in humans in 2002, transcatheter aortic valve implantation (TAVI) has become an established treatment for severe AS^[4-7]^.

Coronary artery disease (CAD) and AS share similar associated clinical risk factors, such as older age, male sex, elevated lipoprotein levels, hypertension, and smoking^[8,9]^. The two conditions often concur, and CAD is prevalent in 30.8-78.2% of patients undergoing TAVI^[10]^. Patients with both severe AS and CAD undergoing surgical AVR have worse early and late survival compared with patients with severe AS alone^[11]^. The clinical impact of CAD in patients undergoing TAVI differs in previous reports. In a meta-analysis, Sankaramangalam et al.^[10]^ (2017) found higher one-year mortality in patients with concomitant CAD, while data from the FRench Aortic National CoreValve and Edwards (FRANCE-2) registry showed similar death rates at a three-year follow-up^[12]^. Prior coronary artery bypass grafting (CABG) may unfavourably influence two-year outcome^[13]^, while prior percutaneous coronary intervention (PCI) does not^[14]^. The aim of this study was to evaluate five-year survival in TAVI patients with or without CAD in a single-center setting.

## METHODS

### Study Design and Population

**Fig. 1:**
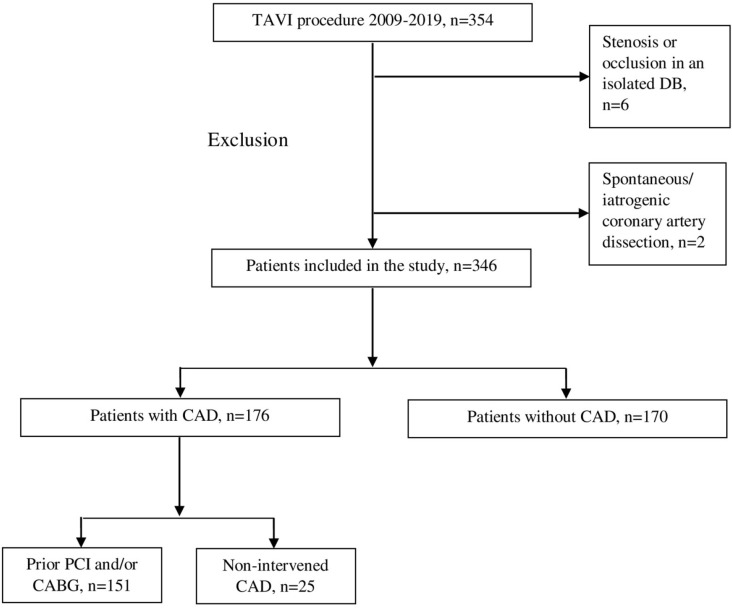
Flow chart of patient inclusion and group division. CABG=coronary artery bypass grafting; CAD=coronary artery disease; DB=diagonal branch; PCI=percutaneous coronary intervention; TAVI=transcatheter aortic valve implantation.

This retrospective observational study included all patients with severe AS undergoing TAVI between September 15, 2009, and November 29, 2019, at Örebro University Hospital (Örebro, Sweden). All included patients had intermediate to high surgical risk. Patients were divided into two groups according to the presence or absence of CAD. All patients underwent preoperative coronary angiography, except for a few cases where computed tomography angiography was performed. Patients with solitary stenosis or occlusion in a minor side branch were excluded from the study. Patients with spontaneous or iatrogenic coronary artery dissection were also excluded (Figure 1). The study was approved by the regional ethical review board (file number: 2019-06442).

### Data Collection

Data were collected from patient files and the Swedish Transcatheter Cardiac Intervention Registry (SWENTRY), a sub-registry of the Swedish Web System for Enhancement and Development of Evidence-Based Care in Heart Disease Evaluated According to Recommended Therapies (or SWEDEHEART). In the SWENTRY, all consecutive patients, from all centers in Sweden, undergoing TAVI are registered. Total follow-up time was defined as the date of the procedure to December 1, 2019. Electronic health records with direct linkage to survival status were used to document survival and cause of death. The primary endpoint, five-year all-cause mortality, was assessed using Cox regression adjusted for age, sex, procedure years, and comorbidities. Presence of comorbidities interacting with CAD, leading to increased five-year all-cause mortality, was evaluated with interaction tests. The secondary endpoint was in-hospital complications.

### Statistical Analysis

Differences in continuous baseline characteristics between the CAD and non-CAD groups were tested with unpaired *t*-test, and ordinal scale characteristics with Mann-Whitney U test. Non-ordered categorical baseline characteristics and number of complications were analysed with chi-square test or Fisher’s exact test where appropriate. Unadjusted Kaplan-Meier and Cox regression analyses were used to visualize and evaluate time to mortality between the CAD and non-CAD groups. The patients were followed up until five years after the procedure, with no censored cases. Crude mortality rates per 1,000 person-years were presented, and adjusted Cox regression was performed in three models. The first model was adjusted for age in five-year categories (< 70, 70-74, 75-79, 80-84, and ≥ 85 years), sex, and procedure year as a categorical variable collapsing years 2009-2012 and 2013-2014 because of sparse data. The second model further adjusted for estimated glomerular filtration rate (eGFR) < 50 ml/min/1.73 m^2^, chronic lung disease (CLD), and pulmonary hypertension. The third model further adjusted for peripheral arterial disease (PAD) and left ventricular ejection fraction (LVEF) < 50%. The variables included in the three models were retrieved from the European System for Cardiac Operative Risk Evaluation (EuroSCORE). The purpose of dividing these variables into three models was to detect and eventually avoid over adjusting for risk factors. The potential effect modification of each adjusted variable described above on mortality, by CAD group, was evaluated with interaction tests. When non-proportional hazards were present, tested on the basis of Schoenfeld residuals, risk time was split at one year and time-dependent Cox regression was used. Cox regression gives hazard ratios (HRs) with 95% confidence intervals (CIs) as association measures. A *P*-value < 0.05 was regarded as statistically significant. All statistical computations were performed with STATA release 14 (StataCorp College Station, Texas, United States of America; www.stata.com) or IBM Corp. Released 2017, IBM SPSS Statistics for Windows, version 25.0, Armonk, NY: IBM Corp.

### Definitions

CAD was defined as either the presence of a significant stenosis or occlusion in one or more coronary arteries or prior PCI and/or CABG, in line with the SYNergy between percutaneous coronary intervention with TAXus and cardiac surgery (SYNTAX) trial.

Vascular complications (major and minor) and stroke were defined according to the Valve Academic Research Consortium-2 (or VARC-2) consensus document.

The analysed comorbidities followed the categorization and definitions included in the EuroSCORE I risk model. Extracardiac arteriopathy is referred to as “peripheral arterial disease (PAD)” in our study.

## RESULTS

In total, 176/346 (50.9%) patients had concomitant CAD and AS, while 170/346 (49.1%) had AS only. Prior PCI and/or CABG was performed in 151/346 (43.6%) patients, and 25 patients had CAD without prior coronary intervention. Baseline clinical characteristics are shown in Table 1. Significantly more patients in the CAD-AS group were males, and had PAD, a LVEF < 50%, hypertension, or a higher logistic EuroSCORE I. In the non-CAD group, more patients had CLD and elevated pulmonary artery pressure.

**Table 1:** Patients’ characteristics and comorbidities of the coronary artery disease (CAD) and non-CAD groups at baseline.

Patients’ characteristics	CAD group (n=176)	Non-CAD group (n=170)	*P*-value
Age, years, mean (SD)	80.5 (5.9)	79.7 (7.5)	0.23
Men, n (%)	103 (58.5)	64 (37.6)	< 0.001
Surgery year, n (%)			0.015
2009-2012	28 (15.9)	12 (7.1)	
2013-2014	20 (11.4)	22 (12.9)	
2015	27 (15.3)	12 (7.1)	
2016	18 (10.2)	21 (12.3)	
2017	19 (10.8)	23 (13.5)	
2018	33 (18.8)	34 (20.0)	
2019	31 (17.6)	46 (27.1)	
eGFR < 50, n (%)	44 (25.0)	34 (20.0)	0.27
CLD, n (%)	20 (11.4)	46 (27.1)	< 0.001
Pulmonary hypertension, n (%)	n=157	n=153	0.019
≤ 30 (normal)	63 (40.1)	41 (26.8)	
31-55	76 (48.4)	98 (64.0)	
> 55	18 (11.5)	14 (9.2)	
PAD, n (%)	39 (22.2)	17 (10.0)	0.002
LVEF < 50%, n (%)	59 (33.5)	33 (19.4)	0.003
Body mass index, mean (SD)	27.0 (5.7)	27.1 (5.5)	0.90
Body mass index, WHO classification			0.32
< 18.5 (underweight), n (%)	2 (1.1)	7 (4.1)	
18.5-24.9 (normal weight), n (%)	71 (40.3)	60 (35.3)	
25.0-29.9 (pre-obesity), n (%)	61 (34.7)	56 (32.9)	
30.0-34.9 (obesity class I), n (%)	29 (16.5)	36 (21.2)	
≥ 35.0 (obesity class II or III), n (%)	13 (7.4)	11 (6.5)	
Smoking	n=159	n=153	0.24
Active smoker, n (%)	14 (8.8)	12 (7.8)	
Ex-smoker, n (%)	76 (47.8)	60 (39.2)	
Never smoked, n (%)	69 (43.4)	81 (52.9)	
Myocardial infarction ≤ 3 months, n (%)	12 (6.8)	-	
Hypertension, n (%)	154 (87.5)	129 (75.9)	0.005
Diabetes, n (%)	55 (31.2)	46 (27.1)	0.39
Insulin, n (%)	22 (12.5)	20 (11.8)	0.83
Prior CABG, n (%)	65 (36.9)	-	-
Prior PCI, n (%)	107 (60.8)	-	-
Non-intervened CAD, n (%)	25 (14.2)	-	-
Prior BAV, n (%)	6 (3.4)	2 (1.2)	0.28
Stroke, n (%)	22 (12.5)	18 (10.6)	0.58
Critical preoperative state, n (%)	3 (1.7)	4 (2.4)	0.72
Acute surgery, n (%)	0 (0.0)	0 (0.0)	-
Atrial fibrillation, n (%)	62 (35.2)	70 (41.2)	0.26
Dialysis, n (%)	1 (0.6)	3 (1.8)	0.36
NYHA function class III or IV, n (%)	160 (90.9)	150 (88.2)	0.42
Logistic EuroSCORE I	n=137	n=138	< 0.001
mean % (SD)	20.6 (14.2)	13.7 (8.7)	

BAV=balloon aortic valvuloplasty; CABG=coronary artery bypass grafting; CLD=chronic lung disease; eGFR=estimated glomerular filtration rate; EuroSCORE=European System for Cardiac Operative Risk Evaluation; LVEF=left ventricular ejection fraction; NYHA=New York Heart Association; PAD=peripheral arterial disease; PCI=percutaneous coronary intervention; SD=standard deviation; WHO=World Health Organization

### In-Hospital Complications

In relation to the TAVI procedure, 25/176 (14.2%) and 18/170 (10.6%) patients experienced complications (vascular, new permanent pacemaker, stroke, or death) in the CAD and non-CAD groups, respectively (*P*=0.308).

### Five-Year All-Cause Mortality and Cause of Death

**Fig. 2:**
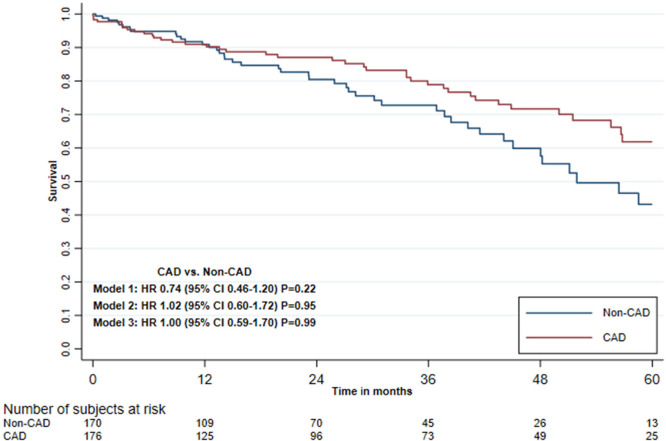
Unadjusted Kaplan-Meier curve showing the five-year survival for transcatheter aortic valve implantation patients with or without coronary artery disease (CAD). CI=confidence interval; HR=hazard ratio.

The mean total follow-up time was 2.2±1.6 years. Among patients with surgery before December 1, 2014, with possible five-year follow-up, the all-cause mortality was 42/80 (52.5%); 23/48 (47.9%) in the CAD group and 19/32 (59.4%) in the non-CAD group. Cardiac death was numerically more common in the CAD group (13/23) than in the non-CAD group (8/19), but the difference was not statistically significant. Cause of death was missing in two patients in each group. A Kaplan-Meier curve illustrates the cumulative, unadjusted five-year survival in the CAD and non-CAD groups (Figure 2) in all 346 patients. Cox regression revealed no differences in five-year all-cause mortality between patients with and patients without CAD in the three different adjustment models (Table 2). In the third adjusted model, HR was 1.00 (95% CI 0.59-1.70), but patients with eGFR < 50 ml/min/1.73 m2 (HR 1.75 [95% CI 1.04-2.94]) and CLD (HR 2.20 [95% CI 1.26-3.84]) had a significantly increased mortality risk (Table 2).

**Table 2:** Cox regression analysis of mortality and comorbidities in the coronary artery disease (CAD) group and the non-CAD group.

0-60 months	n	Events	Rate	Model 1	Model 2	Model 3
				HR (95% CI)	HR (95% CI)	HR (95% CI)
Non-CAD group	170	43	10.8	1.0	1.0	1.0
CAD group	176	39	7.4	0.74 (0.46-1.20)	1.02 (0.60-1.72)	1.00 (0.59-1.70)
				*P*=0.22	*P*=0.95	*P*=0.99
eGFR < 50						
No	268	58	7.9	1.0	1.0	1.0
Yes	78	24	12.2	1.50 (0.92-2.43)	1.78 (1.07-2.98)	1.75 (1.04-2.94)
				*P*=0.10	*P*=0.027	*P*=0.034
CLD						
No	280	56	7.5	1.0	1.0	1.0
Yes	66	26	14.6	2.00 (1.22-3.27)	2.26 (1.31-3.90)	2.20 (1.26-3.84)
				*P*=0.006	*P*=0.003	*P*=0.006
Pulmonary hypertension						
≤ 30 (normal)	104	19	6.5	1.0	1.0	1.0
31-55	174	44	9.8	1.37 (0.77-2.45)	1.34 (0.74-2.40)	1.30 (0.72-2.35)
				*P*=0.29	*P*=0.33	*P*=0.38
> 55	32	11	10.9	1.27 (0.57-2.45)	1.30 (0.58-2.90)	1.21 (0.52-2.81)
				*P*=0.56	*P*=0.52	*P*=0.66
PAD						
No	290	64	8.3	1.0		1.0
Yes	56	18	11.5	1.18 (0.67-2.05)		1.07 (0.56-2.08)
				*P*=0.57		*P*=0.83
LVEF < 50%						
No	254	55	7.8	1.0		1.0
Yes	92	27	11.9	1.58 (0.97-2.58)		1.16 (0.66-2.03)
				*P*=0.063		*P*=0.60

Events: Number of deaths

Rate: Crude number of deaths per 1,000 person-years

Model 1: Adjusted for age, sex, and year of surgery

Model 2: Adjusted for age, sex, year of surgery, eGFR < 50, chronic pulmonary disease, and pulmonary hypertension

Model 3: Adjusted for age, sex, year of surgery, eGFR < 50, chronic pulmonary disease, pulmonary hypertension, PAD, and LVEF < 50

CI=confidence interval; CLD=chronic lung disease; eGFR=estimated glomerular filtration rate; HR=hazard ratio; LVEF=left ventricular ejection fraction; PAD=peripheral arterial disease

### Interaction Between Coronary Artery Disease and Comorbidities on Five-Year Mortality

**Fig. 3:**
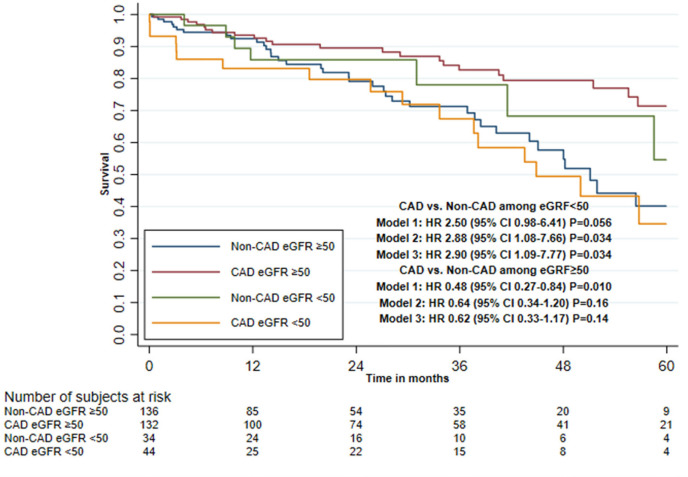
Five-year survival after transcatheter aortic valve implantation in patients with or without concomitant coronary artery disease (CAD), stratified by renal function (Kaplan-Meier estimate). CI=confidence interval; eGFR=estimated glomerular filtration rate; HR=hazard ratio.

**Fig. 4:**
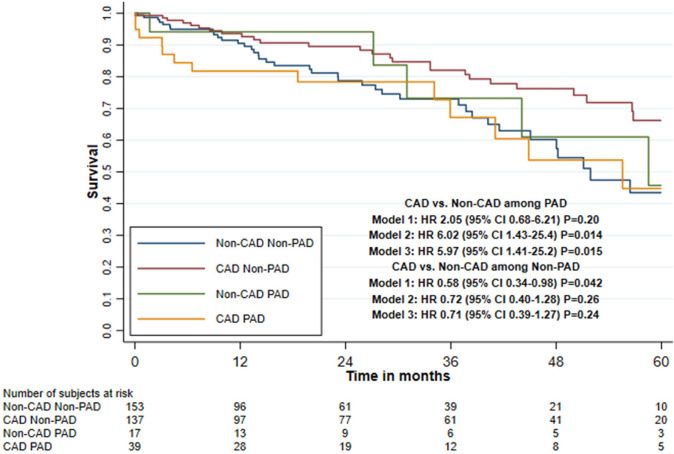
Five-year survival after transcatheter aortic valve implantation in patients with or without concomitant coronary artery disease (CAD), stratified by peripheral arterial disease (PAD) (Kaplan-Meier estimate). CI=confidence interval; HR=hazard ratio.

**Fig. 5:**
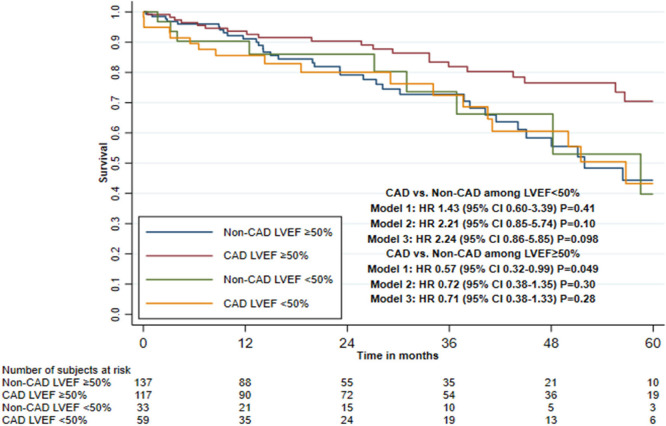
Five-year survival after transcatheter aortic valve implantation in patients with or without concomitant coronary artery disease (CAD), stratified by left ventricular ejection fraction (LVEF) (Kaplan-Meier estimate). CI=confidence interval; HR=hazard ratio.

**Supplementary Fig. 1:**
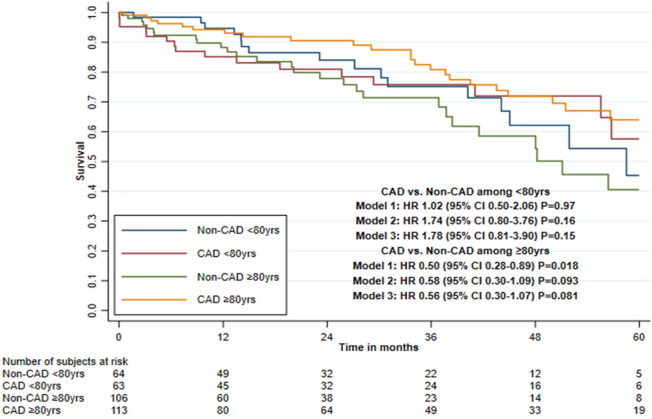
Five-year survival after transcatheter aortic valve implantation in patients with or without concomitant coronary artery disease (CAD), stratified by age (Kaplan-Meier estimate). CI=confidence interval; HR=hazard ratio.

**Supplementary Fig. 2:**
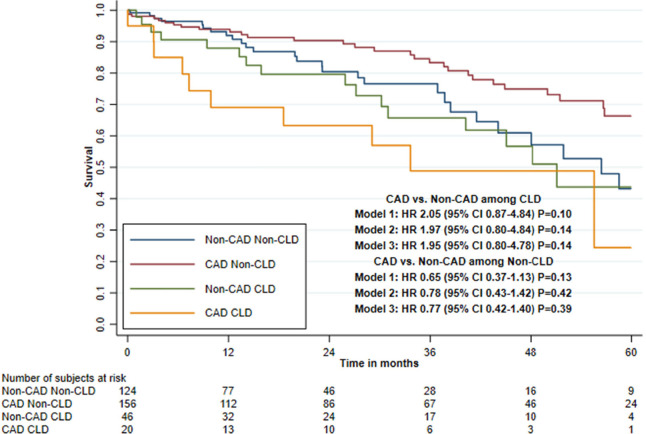
Five-year survival after transcatheter aortic valve implantation in patients with or without concomitant coronary artery disease (CAD), stratified by chronic lung disease (CLD) (Kaplan-Meier estimate). CI=confidence interval; HR=hazard ratio.

Impaired renal function and presence of PAD showed a statistically significant interaction effect with CAD and mortality in all three adjusted models, with LVEF < 50% and age (≤ 79 *vs.* ≥ 80 years) only in the second and third adjusted models and with CLD only in the first adjusted model (Table 3, Figures 3 to 5, and Supplementary Figures 1 and 2).

**Table 3:** Cox regression analysis of mortality stratified to different comorbidities and age in the coronary artery disease (CAD) group *vs.* the non-CAD group.

0-60 months	n	Events	Rate	Model 1	Model 2	Model 3
				HR (95% CI)	HR (95% CI)	HR (95% CI)
				*P*-value	*P*-value	*P*-value
CAD status combined with eGFR						
Normal eGFR						
Non-CAD group	136	36	11.5	1.0	1.0	1.0
CAD group	132	22	5.2	0.48 (0.27-0.84)	0.64 (0.34-1.20)	0.62 (0.33-1.17)
				*P*=0.010	*P*=0.16	*P*=0.14
eGFR < 50						
Non-CAD group	34	7	8.1	1.0	1.0	1.0
CAD group	44	17	15.4	2.50 (0.98-6.41)	2.88 (1.08-7.66)	2.90 (1.09-7.77)
				*P*=0.056	*P*=0.034	*P*=0.034
Interaction tests^1^				*P*=0.002	*P*=0.008	*P*=0.007
CAD status combined with CLD						
No CLD						
Non-CAD group	124	27	9.9	1.0	1.0	1.0
CAD group	156	29	6.1	0.65 (0.37-1.13)	0.78 (0.43-1.42)	0.77 (0.42-1.40)
				*P*=0.13	*P*=0.42	*P*=0.39
Presence of CLD						
Non-CAD group	46	16	12.7	1.0	1.0	1.0
CAD group	20	10	19.1	2.05 (0.87-4.84)	1.97 (0.80-4.84)	1.95 (0.80-4.78)
				*P*=0.10	*P*=0.14	*P*=0.14
Interaction tests^1^				*P*=0.027	*P*=0.088	*P*=0.083
CAD status combined with PAD						
No PAD						
Non-CAD group	153	38	11.0	1.0	1.0	1.0
CAD group	137	26	6.1	0.58 (0.34-0.98)	0.72 (0.40-1.28)	0.71 (0.39-1.27)
				*P*=0.042	*P*=0.26	*P*=0.24
Presence of PAD						
Non-CAD group	17	5	9.6	1.0	1.0	1.0
CAD group	39	13	12.5	2.05 (0.68-6.21)	6.02 (1.43-25.4)^2^	5.97 (1.41-25.2)^2^
				*P*=0.20	*P*=0.014^2^	*P*=0.015^2^
Interaction tests^1^				*P*=0.031	*P*=0.003	*P*=0.003
1-year follow-up						
No PAD						
Non-CAD group	153	12	8.2	1.0	1.0	1.0
CAD group	137	8	5.6	0.69 (0.28-1.71)	0.77 (0.30-1.94)	0.76 (0.30-1.92)
				*P*=0.42	*P*=0.58	*P*=0.56
Patients with PAD						
Non-CAD group	17	1	5.7	1.0	1.0	1.0
CAD group	39	7	18.1	3.84 (0.46-31.9) *P*=0.21	NE	NE
CAD status combined with LVEF						
Patients with LVEF ≥ 50%						
Non-CAD group	137	34	10.7	1.0	1.0	1.0
CAD group	117	21	5.5	0.57 (0.32-0.99)	0.72 (0.38-1.35)	0.71 (0.38-1.33)
				*P*=0.049	*P*=0.30	*P*=0.28
Patients with LVEF < 50%						
Non-CAD group	33	9	11.0	1.0	1.0	1.0
CAD group	59	18	12.4	1.43 (0.60-3.39)	2.21 (0.85-5.74)	2.24 (0.86-5.85)
				*P*=0.41	*P*=0.10	*P*=0.098
Interaction tests^1^				*P*=0.061	*P*=0.037	*P*=0.034
CAD status combined with age						
< 80-year-old patients						
Non-CAD group	64	16	8.9	1.0	1.0	1.0
CAD group	63	16	8.9	1.02 (0.50-2.06)^2^	1.74 (0.80-3.76)^2^	1.78 (0.81-3.90)^2^
				*P*=0.97^2^	*P*=0.16^2^	*P*=0.15^2^
≥ 80-year-old patients						
Non-CAD group	106	27	12.3	1.0	1.0	1.0
CAD group	113	23	6.6	0.50 (0.28-0.89)	0.58 (0.30-1.09)	0.56 (0.30-1.07)
				*P*=0.018	*P*=0.093	*P*=0.081
Interaction tests^1^				*P*=0.12	*P*=0.025	*P*=0.021
1-year follow-up						
< 80-year-old patients						
Non-CAD group	52	3	4.3	1.0	1.0	1.0
CAD group	53	9	14.1	3.15 (0.85-11.7)	6.27 (1.33-29.6)	6.19 (1.30-29.5)
				*P*=0.086	*P*=0.020	*P*=0.022
≥ 80-year-old patients						
Non-CAD group	73	19	10.6	1.0	1.0	1.0
CAD group	86	6	5.1	0.45 (0.16-1.26)	0.44 (0.14-1.30)	0.43 (0.14-1.28)
				*P*=0.13	*P*=0.14	*P*=0.13

Events: Number of deaths

Rate: Crude number of deaths per 1,000 person-years

Model 1: Adjusted for age, sex, and year of surgery

Model 2: Adjusted for age, sex, year of surgery, eGFR < 50, chronic pulmonary disease, and pulmonary hypertension

Model 3: Adjusted for age, sex, year of surgery, eGFR < 50, chronic pulmonary disease, pulmonary hypertension, PAD, and LVEF < 50

1 Interaction tests were conducted if the CAD and non-CAD groups’ association with mortality was statistically significantly different in comorbidity and age sub-groups

2 Non-proportional hazards present at 5 years and 1 year were analysed

CI=confidence interval; CLD=chronic lung disease; eGFR=estimated glomerular filtration rate; HR=hazard ratio; LVEF=left ventricular ejection fraction; NE=no estimate (because of sparse data); PAD=peripheral arterial disease

Among patients with impaired renal function, the HR was 2.90 (95% CI 1.09-7.77) when comparing the CAD and non-CAD group in the third adjusted model. Among patients with PAD, the HR was 5.97 (95% CI 1.41-25.2); however, the hazard was non-proportional and because of few mortality cases it was not possible to evaluate only the first year of follow-up. Among patients with LVEF < 50%, the HR was 2.24 (95% CI 0.86-5.85) when comparing the CAD and non-CAD group. Among patients aged < 80 years, the HR was 1.78 (95% CI 0.81-3.90) but the hazard was non-proportional, and for the first year of follow-up, the HR was 6.19 (95% CI 1.30-29.5). Among patients with CLD, the HR was 1.95 (95% CI 0.80-4.78) (Table 3).

## DISCUSSION

In this single-center observational study, we did not find differences in five-year mortality between patients with AS and CAD, and patients with AS alone undergoing TAVI. However, renal impairment, PAD, LVEF < 50%, and age ≥ 80 years in addition to CAD were associated with significantly higher five-year mortality.

The five-year all-cause mortality of 52.5% in our study is in line with previous studies reporting a mortality rate between 41.0% and 67.8%^[4,15]^. The proportion of CAD patients, 50.9%, is also consistent with a previously conducted meta-analysis^[10]^. At baseline, patients with CAD had a significantly higher logistic EuroSCORE I and more cardiovascular risk factors. Despite this, the CAD group had a similar five-year all-cause mortality.

Most previous studies investigating the impact of CAD on TAVI outcomes report follow-up times of no longer than three years. The populations and definitions of CAD in these studies have differed. Kawashima et al.^[13]^ found that TAVI patients with previous CABG had a higher rate of two-year all-cause mortality and cardiovascular death. However, in their study, CAD was also present in the group without prior CABG. Results from the FRANCE-2 registry showed that CAD was not associated with increased mortality at 30 days or three years but the authors found that stenosis of the left anterior descending coronary artery was associated with higher three-year mortality^[12]^. One important factor to note is that patients with prior CABG were excluded from their study, which may explain why the prevalence of CAD was lower, at 36%. Two meta-analyses on the subject reached different conclusions: Sankaramangalam et al.^[10]^ in 2017 studied the impact of CAD (n=3,899) in patients (n=8,013) undergoing a TAVI procedure and showed that the CAD group had a significantly lower survival at one year. In the same year, Kotronias et al.^[14]^ investigated the effect of previous PCI (n=983) *vs.* no previous PCI in TAVI patients (n=3,858) on one-year all-cause mortality. They found no differences between groups.

In one study, impaired renal function (eGFR < 30 ml/min/1.73 m^2^) was associated with increased one-year mortality after TAVI^[16]^. In another report, impaired renal function with eGFR < 60 ml/min/1.73 m^2^ was not associated with increased death rates at one year after TAVI^[17]^. Impaired renal function in our study (eGFR < 50 ml/min/1.73 m^2^) was not associated with increased five-year mortality but the interaction between CAD and impaired renal failure was significant and associated with higher five-year mortality.

The total prevalence of PAD was 22.1% and 10.0% in the CAD and non-CAD groups, respectively. This is in line with previous studies reporting a prevalence of 19.2-25.1%^[18,19]^. PAD has been associated with increased early (< 30 days) and late (> 12 months) mortality after TAVI^[18,20]^. Such association was only seen when the interaction between PAD and CAD was analysed in our study. There were more patients with PAD in the CAD than in the non-CAD group, which was unsurprising given the overlap of risk factors.

Pulmonary hypertension and impaired LVEF in patients with AS are common indicators of advanced disease usually resulting in poor prognosis^[21,22]^. Contradicting other reports^[21,23]^, in our study neither of these comorbidities alone had an impact on five-year mortality. CLD was the only isolated comorbidity associated with higher five-year mortality after TAVI. This finding is supported by previous studies^[24]^ and may prove helpful in future patient selection.

Until recently, and before the publication of the low-risk TAVI trials^[6,7]^, TAVI was mainly reserved for elderly patients with intermediate or high operative risk. With this in mind, we studied the interaction between CAD and age ≥ 80 years and found higher five-year mortality. Our study is a small contribution to the current evidence gap of TAVI patients with CAD and is in line with an ongoing trial - the COMPLETE TAVR (ClinicalTrials.gov: NCT04634240). Additionally, our data shows similar results as the newly published study from the percutAneous Coronary inTervention prIor to transcatheter aortic VAlve implantation (or ACTIVATION) trial; similar observed rates of death and rehospitalizations at 1 year between PCI and no PCI prior to TAVI^[25]^, albeit their time frame of one year differs from our five years.

### Limitations

There is a risk for bias in this observational study, mainly due to the selection and classification of patients, potential confounding factors not accounted for in our analysis in addition to bias for missing data. Missing data in the registry were addressed through reviewing and adding data from the individual patient’s electronic health records. However, not all missing data were accounted for using this method. As this was a single-center study, the number of participants was relatively small, and the number of patients having a follow-up of five years or longer was limited to a total of 80 patients. Unfortunately, SYNTAX score was not registered for all the TAVI patients with CAD.

## CONCLUSION

Overall, five-year mortality did not differ between patients with and without CAD undergoing TAVI. However, patients with CAD and additional risk factors, such as impaired renal function, PAD, or reduced LVEF, had significantly higher five-year all-cause mortality.

## Impact On Daily Practice

TAVI has evolved as a new standard in the treatment of patients with severe AS. Understanding the interaction between concomitant diseases and survival is vital in future patient selection.

## Data Availability

The data that support the findings of this study are available on request from the corresponding author. The data are not publicly available due to privacy or ethical restrictions.

## Abbreviations, Acronyms & Symbols

AS: = Aortic stenosis
AVR: = Aortic valve replacement
BAV: = Balloon aortic valvuloplasty
CABG: = Coronary artery bypass grafting
CAD: = Coronary artery disease
CI: = Confidence interval
CLD: = Chronic lung disease
DB: = Diagonal branch
eGFR: = Estimated glomerular filtration rate
EuroSCORE: = European System for Cardiac Operative Risk Evaluation
FRANCE-2: = FRench Aortic National CoreValve and Edwards
HR: = Hazard ratio
LVEF: = Left ventricular ejection fraction
NE: = No estimate (because of sparse data)
NYHA: = New York Heart Association
PAD: = Peripheral arterial disease
PCI: = Percutaneous coronary intervention
SD: = Standard deviation
SWENTRY: = Swedish Transcatheter Cardiac Intervention Registry
SYNTAX: SYNergy between percutaneous coronary intervention with TAXus and cardiac surgery
TAVI: = Transcatheter aortic valve implantation
WHO: = World Health Organization
